# Poly[bis­(μ-2,6-dimethyl­pyridinium-3,5-dicarboxyl­ato-*κ* ^2^ *O* ^3^:*O* ^5^)copper(II)]

**DOI:** 10.1107/S1600536808035873

**Published:** 2008-11-08

**Authors:** Hong-Kun Zhang, Yu-Hong Du, Tao Jiang, Bai-Yan Li, Guang-Feng Hou

**Affiliations:** a Department of Food and Environmental Engineering, Heilongjiang East College, Harbin 150086, People’s Republic of China; b College of Chemistry and Materials Science, Heilongjiang University, Harbin 150080, People’s Republic of China

## Abstract

In the title coordination polymer, [Cu(C_9_H_8_NO_4_)_2_]_*n*_, the Cu atom, located on a twofold rotation axis, is four coordinate in a distorted square-planar environment. Each 2,6-dimethyl­pyridinium-3,5-dicarboxyl­ate anion bridges two Cu atoms, forming a two-dimensional coordination polymer. A three-dimensional supra­molecular network is built from N—H⋯O hydrogen bonds involving the pyridinium NH and the carboxyl COO groups.

## Related literature

For the synthesis of 2,6-dimethyl­pyridine-3,5-dicarboxylic acid, see: Checchi *et al.* (1959 ▶). For the crystal structures of some of its metal complexes, see: Gao *et al.* (2007 ▶); Shi *et al.* (2007 ▶); Zeng *et al.* (2000 ▶, 2002 ▶).
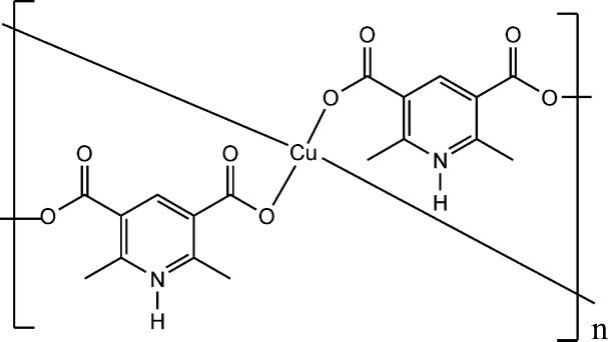


## Experimental

### 

#### Crystal data

[Cu(C_9_H_8_N_2_O_4_)_2_]
*M* *_r_* = 451.87
Orthorhombic, 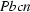

*a* = 8.2003 (16) Å
*b* = 16.234 (3) Å
*c* = 13.708 (3) Å
*V* = 1824.9 (6) Å^3^
*Z* = 4
Mo *K*α radiation
μ = 1.25 mm^−1^
*T* = 291 (2) K
0.26 × 0.24 × 0.19 mm

#### Data collection

Rigaku R-AXIS RAPID diffractometer
Absorption correction: multi-scan (*ABSCOR*; Higashi, 1995 ▶) *T*_min_ = 0.733, *T*_max_ = 0.801
16747 measured reflections
2097 independent reflections
1754 reflections with *I* > 2σ(*I*)
*R*_int_ = 0.051

#### Refinement

*R*[*F* ^2^ > 2σ(*F* ^2^)] = 0.033
*wR*(*F* ^2^) = 0.092
*S* = 1.09
2097 reflections
138 parameters
H atoms treated by a mixture of independent and constrained refinement
Δρ_max_ = 0.41 e Å^−3^
Δρ_min_ = −0.29 e Å^−3^

### 

Data collection: *RAPID-AUTO* (Rigaku, 1998 ▶); cell refinement: *RAPID-AUTO*; data reduction: *CrystalStructure* (Rigaku/MSC, 2002 ▶); program(s) used to solve structure: *SHELXS97* (Sheldrick, 2008 ▶); program(s) used to refine structure: *SHELXL97* (Sheldrick, 2008 ▶); molecular graphics: *SHELXTL* (Sheldrick, 2008 ▶); software used to prepare material for publication: *SHELXL97*.

## Supplementary Material

Crystal structure: contains datablocks global, I. DOI: 10.1107/S1600536808035873/ng2511sup1.cif

Structure factors: contains datablocks I. DOI: 10.1107/S1600536808035873/ng2511Isup2.hkl

Additional supplementary materials:  crystallographic information; 3D view; checkCIF report

## Figures and Tables

**Table 1:** Hydrogen-bond geometry (Å, °)

*D*—H⋯*A*	*D*—H	H⋯*A*	*D*⋯*A*	*D*—H⋯*A*
N1—H8⋯O3^i^	0.82 (3)	1.88 (3)	2.698 (2)	177 (3)

Symmetry code: (i) 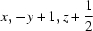.

**Fig. 1.**
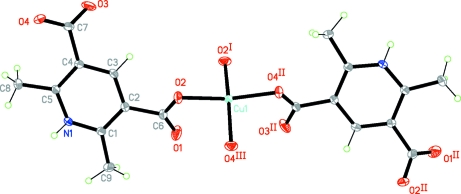
The molecular structure of (I), showing displacement ellipsoids at the 30% probability level for non-H atoms. [symmetry codes: (I): -x - 1, -y + 1, -z; (II): x - 1, 3/2 - y, -1/2 + z; (III): x, -1/2 - y, -1/2 + z]

**Fig. 2.**
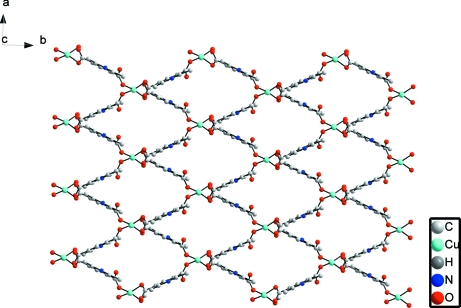
Part of the polymeric structure of (I), showing a two-dimensional network.

## supplementary crystallographic information

### Comment

To the best of our knowledge, there have been few reports to date on the crystal structure of 2,6-dimethylpyrine-3,5-dicarboxylic acid ligand (Zeng *et al.*, 2000; Zeng *et al.*, 2002: Gao *et al.*, 2007). The crystal structure of 2,6-dimethylpyridinium-3,5-dicarboxylate ligand and Cu atom complex have been reported, namely *trans*-tetraaquabis (2,6-dimethylpyrinium-3,5-dicarboxylate)cooper(II) tetrahydrate, which is a discrete compound (Shi *et al.*, 2007). In this paper, we report the new two-dimensional title complex, (I), synthesized by the recation of 2,6-dimethylpyrine-3,5-dicarboxylic acid and copper(II) dinitrate in methanol solution.

In the title compound, (Fig. 1), the Cu atom is located on a twofold rotation axis is four coordinated in a square environment that is formed by four carboxylate O atoms from four 2,6-dimethylpyridinium-3,5- dicarboxylate ligands. Each 2,6-dimethylpyridinium-3,5-dicarboxylate ligand bridges two Cu atom to form a two-dimensional supramolecular network parallel the *ab* plane (Fig. 2). In addition, N1—H8···O3^i^ hydrogen bonds link these adjacent plane into a three-dimensional supramolecular network (Table 1).

### Experimental

2,6-Dimethylpyridine-3,5-dicarboxylic acid was prepared by basic hydrolysis of diethyl 2,6-dimethylpyridine-3,5-dicarboxylate, prepared according to Checchi (1959). Diethyl 2,6-dimethylpyridine-3,5-dicarboxylate (25.1 g, 0.1 mol) and potassium hydroxide (13.44 g, 0.24 mol) were dissolved in 150 ml e thanol and 150 ml water mixed solution, then stirred for three hours under reflux conditions. 10.5 g 2,6-Dimethylpyridine-3,5-dicarboxylic acid, a white precipitate, formed by adjusting pH of solution to 3 with 0.1 *M* HCl after evaporation of ethanol.

The complex (I) was synthesized with coppert(II) dinitrate (0.368 g, 2 mmol) and 2,6-dimethylpyridine-3,5-dicarboxylic acid (0.390 g, 2 mmol) were dissolved in methanol and the pH was adjusted to 6 with 0.01*M* sodium hydroxide. Black crystals were separated from the filtered solution after several days.

### Refinement

H atoms bound to C atoms were placed in calculated positions and treated as riding on their parent atoms, with C—H = 0.93 Å, 0.97 Å for aromatic and methyl H atoms respectively; *U*_iso_(H) was set to = 1.2*U*_eq_ of the carrier atom (1.5 *U*_eq_ for methyl H atoms). The H8 atoms bond to N1 atoms were located in a difference Fourier map and refined isotropically.

### Crystal data

**Table:**

[Cu(C_9_H_8_N_2_O_4_)_2_]	*F*_000_ = 924
*M**_r_* = 451.87	*D*_x_ = 1.645 Mg m^−^^3^
Orthorhombic, *P**b**c**n*	Mo *K*α radiation λ = 0.71073 Å
Hall symbol: -P 2n 2ab	Cell parameters from 12587 reflections
*a* = 8.2003 (16) Å	θ = 3.2–27.5º
*b* = 16.234 (3) Å	µ = 1.25 mm^−^^1^
*c* = 13.708 (3) Å	*T* = 291 (2) K
*V* = 1824.9 (6) Å^3^	Bluck, black
*Z* = 4	0.26 × 0.24 × 0.19 mm

### Data collection

**Table:**

Rigaku R-AXIS RAPID diffractometer	2097 independent reflections
Radiation source: fine-focus sealed tube	1754 reflections with *I* > 2σ(*I*)
Monochromator: graphite	*R*_int_ = 0.051
*T* = 291(2) K	θ_max_ = 27.5º
ω scan	θ_min_ = 3.2º
Absorption correction: Multi-scan(ABSCOR; Higashi, 1995)	*h* = −10→10
*T*_min_ = 0.733, *T*_max_ = 0.801	*k* = −21→21
16747 measured reflections	*l* = −17→17

### Refinement

**Table:**

Refinement on *F*^2^	Secondary atom site location: difference Fourier map
Least-squares matrix: full	Hydrogen site location: inferred from neighbouring sites
*R*[*F*^2^ > 2σ(*F*^2^)] = 0.033	H atoms treated by a mixture of independent and constrained refinement
*wR*(*F*^2^) = 0.092	*w* = 1/[σ^2^(*F*_o_^2^) + (0.0491*P*)^2^ + 0.9342*P*] where *P* = (*F*_o_^2^ + 2*F*_c_^2^)/3
*S* = 1.09	(Δ/σ)_max_ = 0.008
2097 reflections	Δρ_max_ = 0.41 e Å^−^^3^
138 parameters	Δρ_min_ = −0.29 e Å^−^^3^
Primary atom site location: structure-invariant direct methods	Extinction correction: none

### Special details

**Table:**

Geometry. All e.s.d.'s (except the e.s.d. in the dihedral angle between two l.s. planes) are estimated using the full covariance matrix. The cell e.s.d.'s are taken into account individually in the estimation of e.s.d.'s in distances, angles and torsion angles; correlations between e.s.d.'s in cell parameters are only used when they are defined by crystal symmetry. An approximate (isotropic) treatment of cell e.s.d.'s is used for estimating e.s.d.'s involving l.s. planes.
Refinement. Refinement of *F*^2^ against ALL reflections. The weighted *R*-factor *wR* and goodness of fit *S* are based on *F*^2^, conventional *R*-factors *R* are based on *F*, with *F* set to zero for negative *F*^2^. The threshold expression of *F*^2^ > σ(*F*^2^) is used only for calculating *R*-factors(gt) *etc*. and is not relevant to the choice of reflections for refinement. *R*-factors based on *F*^2^ are statistically about twice as large as those based on *F*, and *R*- factors based on ALL data will be even larger.

### Fractional atomic coordinates and isotropic or equivalent isotropic displacement parameters (Å^2^)

**Table:**

	*x*	*y*	*z*	*U*_iso_*/*U*_eq_	
C1	0.1149 (2)	0.35544 (11)	0.53558 (13)	0.0216 (4)	
C2	0.1113 (2)	0.33765 (12)	0.43628 (14)	0.0226 (4)	
C3	0.1706 (3)	0.39606 (12)	0.37198 (13)	0.0251 (4)	
H1	0.1677	0.3849	0.3055	0.030*	
C4	0.2345 (3)	0.47081 (12)	0.40328 (13)	0.0225 (4)	
C5	0.2407 (3)	0.48643 (11)	0.50282 (13)	0.0211 (4)	
C6	0.0406 (3)	0.25909 (13)	0.39516 (15)	0.0269 (4)	
C7	0.2938 (3)	0.53108 (12)	0.32739 (14)	0.0257 (4)	
C8	0.3043 (3)	0.56351 (13)	0.54895 (15)	0.0300 (5)	
H5	0.2941	0.5595	0.6186	0.045*	
H6	0.2427	0.6099	0.5259	0.045*	
H7	0.4170	0.5706	0.5320	0.045*	
C9	0.0530 (3)	0.30107 (14)	0.61494 (15)	0.0331 (5)	
H2	0.1078	0.3142	0.6749	0.050*	
H3	0.0736	0.2446	0.5984	0.050*	
H4	−0.0621	0.3094	0.6227	0.050*	
Cu1	0.0000	0.148168 (18)	0.2500	0.02018 (13)	
H8	0.186 (3)	0.4388 (17)	0.621 (2)	0.042 (8)*	
N1	0.1800 (2)	0.42800 (10)	0.56291 (12)	0.0225 (4)	
O1	−0.0813 (2)	0.22887 (11)	0.43090 (13)	0.0466 (5)	
O2	0.1169 (2)	0.23282 (9)	0.32017 (10)	0.0333 (4)	
O3	0.2050 (3)	0.54134 (12)	0.25578 (11)	0.0442 (5)	
O4	0.4295 (2)	0.56494 (9)	0.34300 (11)	0.0337 (4)	

### Atomic displacement parameters (Å^2^)

**Table:**

	*U*^11^	*U*^22^	*U*^33^	*U*^12^	*U*^13^	*U*^23^
C1	0.0243 (10)	0.0224 (9)	0.0180 (9)	0.0001 (8)	−0.0003 (7)	0.0006 (7)
C2	0.0248 (10)	0.0226 (9)	0.0203 (9)	−0.0015 (8)	−0.0012 (7)	−0.0027 (7)
C3	0.0331 (11)	0.0271 (10)	0.0152 (8)	−0.0015 (8)	−0.0020 (7)	−0.0029 (7)
C4	0.0283 (10)	0.0217 (9)	0.0175 (8)	−0.0017 (8)	−0.0009 (7)	0.0015 (7)
C5	0.0239 (10)	0.0207 (9)	0.0186 (9)	0.0000 (8)	−0.0020 (7)	0.0002 (7)
C6	0.0319 (11)	0.0239 (10)	0.0250 (10)	−0.0044 (8)	−0.0064 (8)	−0.0013 (8)
C7	0.0382 (12)	0.0203 (9)	0.0186 (9)	0.0020 (9)	0.0036 (8)	0.0015 (7)
C8	0.0399 (12)	0.0254 (10)	0.0246 (10)	−0.0060 (9)	−0.0031 (8)	−0.0051 (8)
C9	0.0442 (13)	0.0325 (11)	0.0225 (10)	−0.0085 (10)	0.0067 (9)	0.0033 (8)
Cu1	0.0283 (2)	0.01488 (19)	0.01735 (19)	0.000	−0.00329 (12)	0.000
N1	0.0294 (9)	0.0249 (8)	0.0132 (7)	−0.0018 (7)	−0.0006 (6)	−0.0012 (6)
O1	0.0439 (11)	0.0449 (10)	0.0511 (10)	−0.0222 (9)	0.0102 (9)	−0.0123 (8)
O2	0.0440 (9)	0.0283 (7)	0.0277 (7)	−0.0087 (7)	−0.0004 (7)	−0.0095 (6)
O3	0.0507 (11)	0.0592 (12)	0.0228 (8)	−0.0022 (9)	−0.0051 (7)	0.0161 (7)
O4	0.0442 (10)	0.0279 (8)	0.0290 (8)	−0.0094 (7)	0.0034 (7)	0.0081 (6)

### Geometric parameters (Å, °)

**Table:**

C1—N1	1.346 (3)	C7—O4	1.259 (3)
C1—C2	1.392 (3)	C8—H5	0.9600
C1—C9	1.490 (3)	C8—H6	0.9600
C2—C3	1.383 (3)	C8—H7	0.9600
C2—C6	1.510 (3)	C9—H2	0.9600
C3—C4	1.390 (3)	C9—H3	0.9600
C3—H1	0.9300	C9—H4	0.9600
C4—C5	1.389 (3)	Cu1—O2	1.9322 (15)
C4—C7	1.509 (3)	Cu1—O2^i^	1.9322 (15)
C5—N1	1.351 (2)	Cu1—O4^ii^	1.9455 (15)
C5—C8	1.496 (3)	Cu1—O4^iii^	1.9455 (15)
C6—O1	1.216 (3)	N1—H8	0.82 (3)
C6—O2	1.277 (3)	O4—Cu1^iv^	1.9455 (15)
C7—O3	1.234 (3)		
			
N1—C1—C2	117.53 (17)	C5—C8—H6	109.5
N1—C1—C9	116.75 (17)	H5—C8—H6	109.5
C2—C1—C9	125.72 (18)	C5—C8—H7	109.5
C3—C2—C1	118.26 (17)	H5—C8—H7	109.5
C3—C2—C6	118.44 (17)	H6—C8—H7	109.5
C1—C2—C6	123.26 (17)	C1—C9—H2	109.5
C2—C3—C4	122.31 (17)	C1—C9—H3	109.5
C2—C3—H1	118.8	H2—C9—H3	109.5
C4—C3—H1	118.8	C1—C9—H4	109.5
C5—C4—C3	118.47 (17)	H2—C9—H4	109.5
C5—C4—C7	123.17 (17)	H3—C9—H4	109.5
C3—C4—C7	118.36 (17)	O2—Cu1—O2^i^	89.32 (10)
N1—C5—C4	117.21 (17)	O2—Cu1—O4^ii^	165.38 (7)
N1—C5—C8	117.25 (16)	O2^i^—Cu1—O4^ii^	91.17 (7)
C4—C5—C8	125.51 (17)	O2—Cu1—O4^iii^	91.17 (7)
O1—C6—O2	126.3 (2)	O2^i^—Cu1—O4^iii^	165.38 (7)
O1—C6—C2	120.40 (19)	O4^ii^—Cu1—O4^iii^	92.03 (10)
O2—C6—C2	113.20 (18)	C1—N1—C5	126.19 (16)
O3—C7—O4	126.7 (2)	C1—N1—H8	119 (2)
O3—C7—C4	116.5 (2)	C5—N1—H8	115 (2)
O4—C7—C4	116.80 (18)	C6—O2—Cu1	113.26 (14)
C5—C8—H5	109.5	C7—O4—Cu1^iv^	117.02 (14)

Symmetry codes: (i) −*x*, *y*, −*z*+1/2; (ii) *x*−1/2, *y*−1/2, −*z*+1/2; (iii) −*x*+1/2, *y*−1/2, *z*; (iv) *x*+1/2, *y*+1/2, −*z*+1/2.

### Hydrogen-bond geometry (Å, °)

**Table:**

*D*—H···*A*	*D*—H	H···*A*	*D*···*A*	*D*—H···*A*
N1—H8···O3^v^	0.82 (3)	1.88 (3)	2.698 (2)	177 (3)

Symmetry codes: (v) *x*, −*y*+1, *z*+1/2.
